# Supplementary material for: Barriers to conducting implementation science research in Asia: An online survey
Source: PLoS One. 2025 Jun 26;20(6):e0325372. doi: 10.1371/journal.pone.0325372 (PMC12200828; doi:10.1371/journal.pone.0325372)
Supplement: S1 File — (PDF) [file pone.0325372.s001.pdf]

S1 File

Search process and strategy, and study questionnaire

The following concepts were used when conducting the literature search and they were “implementation science”, “Asia”, and the specific countries in the Asian region (United Nations, 2023). Their synonyms, and alternative spelling (see Appendix for the search strategy) were included in the search strategy. Both keywords and MESH terms were also used. Countries such as Taiwan, and Yemen, which are not in the United Nations list was also included as search terms for comprehensiveness.

From the literature search, a total of 2295 articles were retrieved. These articles were screened by title and abstract by the two researchers of this study, with the articles divided (WTT=1156; CHO=1139). Articles assessed were included or excluded from the review according to the inclusion and exclusion criteria. Once the researchers finished their screening, they cross-checked each other assessment for correctness. Discrepancies were resolved through discussions.

From the 2295 articles, 2013 articles were removed for the following reasons: studies conducted in non-Asian settings (n=133); multinational studies including non-Asian setting (n=65); not implementation research (n=1109); not original research (n=358); protocol (n=45); articles from a same study (n=26); same corresponding author who conducted in different study (n=7); duplicates (n=267); not in English language: (n=3). The final number of articles included in this study were 282, and the corresponding authors’ email addresses were extracted.

PubMed, 6 Nov 2023

No	Search term	No
#1	"Implementation Science" [MeSH] OR "Implementation Science" [Title/abstract] OR "Implementation research" [Title/abstract] OR "Knowledge translation" [Title/abstract] OR "Knowledge transfer" [Title/abstract] OR “Translational research” [Title/abstract] OR “Dissemination and implementation research” [Title/abstract] or “D&I research” [Title/abstract]	30,441
#2	"Asia" [MeSH Terms] OR "China" [MeSH Terms] OR "Hong Kong" [MeSH Terms] OR "Japan" [MeSH Terms] OR "Korea" [MeSH Terms] OR "Taiwan" [MeSH Terms] OR "Mongolia" [MeSH Terms] OR "Bangladesh" [MeSH Terms] OR "Bhutan" [MeSH Terms] OR "India" [MeSH Terms] OR "Nepal" [MeSH Terms] OR "Sri Lanka" [MeSH Terms] OR "Brunei" [MeSH Terms] OR "Myanmar" [MeSH Terms] OR "Cambodia" [MeSH Terms] OR "Indonesia" [MeSH Terms] OR "Laos" [MeSH Terms] OR "Philippines" [MeSH Terms] OR "Malaysia" [MeSH Terms] OR "Singapore" [MeSH Terms] OR "Thailand" [MeSH Terms] OR "Timor-Leste" [MeSH Terms] OR "Vietnam" [MeSH Terms] OR "Macau" [MeSH Terms] OR "Bahrain" [MeSH Terms] OR "Armenia" [MeSH Terms] OR "Qatar" [MeSH Terms] OR "Georgia" [MeSH Terms] OR "Kuwait" [MeSH Terms] OR "Oman" [MeSH Terms] OR "Turkmenistan" [MeSH Terms] OR "Lebanon" [MeSH Terms] OR "Kyrgyzstan" [MeSH Terms] OR "Israel" [MeSH Terms] OR "Tajikistan" [MeSH Terms] OR "United Arab Emirates" [MeSH Terms] OR "Azerbaijan" [MeSH Terms] OR "Jordan" [MeSH Terms] OR "Kazakhstan" [MeSH Terms] OR "Syria" [MeSH Terms] OR "Yemen" [MeSH Terms] OR "Uzbekistan" [MeSH	1,059,344

	Terms] OR "Saudi Arabia" [MeSH Terms] OR "Afghanistan" [MeSH Terms] OR "Iraq" [MeSH Terms] OR "Iran" [MeSH Terms] OR "Pakistan" [MeSH Terms] OR “Cyprus” [MeSH Terms] OR “Turkey” [MeSH Terms] OR “Maldives” [MeSH Terms] OR	
#3	"Asia*" [Title/abstract] OR "China" [Title/abstract] OR "Chinese" [Title/abstract] OR "Hong Kong" [Title/abstract] OR "Japan*" [Title/abstract] OR "Korea*" [Title/abstract] OR "Taiwan*" [Title/abstract] OR "Mongolia*" [Title/abstract] OR "Bangla*"[Title/abstract] OR "Bhutan*" [Title/abstract] OR "India*" [Title/abstract] OR "Maldiv*" [Title/abstract] OR "Nepal*" [Title/abstract] OR "Sri Lanka*" [Title/abstract] OR "Brunei*" [Title/abstract] OR "Myanmar*" [Title/abstract] OR "Burm*" [Title/abstract] OR "Cambodia*" [Title/abstract] OR "Indonesia*" [Title/abstract] OR "Laos" [Title/abstract] OR "Lao People’s Democratic Republic" [Title/abstract] OR "Laotian*" [Title/abstract] OR "Lao PDR" [Title/abstract] OR "Philippin*"[Title/abstract] OR "Phillippin*" [Title/abstract] OR "Filipin*" [Title/abstract] OR "Malay*" [Title/abstract] OR "Singapore*" [Title/abstract] OR "Thai*" [Title/abstract] OR "Timor Leste*" [Title/abstract] OR "Timor*" [Title/abstract] OR "Timor-Leste*" [Title/abstract] OR "East Timor" [Title/abstract] OR "Vietnam*" [Title/abstract] OR "Viet Nam" [Title/abstract] OR "Macau*" [Title/abstract] OR "Macao*" [Title/abstract] OR "Macanese*" [Title/abstract] OR "Bahrain*" [Title/abstract] OR "Armenia*" [Title/abstract] OR "Qatar*" [Title/abstract] OR "Georgia*" [Title/abstract] OR "Kuwait*" [Title/abstract] OR "Oman*" [Title/abstract] OR "Palestin*" [Title/abstract] OR "Turkmen*" [Title/abstract] OR "Lebanon" [Title/abstract] OR "Leban*" [Title/abstract] OR "Kyrgyz*" [Title/abstract] OR "Israel*" [Title/abstract] OR "Tajikistan" [Title/abstract] OR "Tadzhikistan" [Title/abstract] OR "Tajik*" [Title/abstract] OR "United Arab Emirates" [Title/abstract] OR "Emirat*" [Title/abstract] OR "UAE" [Title/abstract] OR "Azerbaijan*" [Title/abstract] OR "Azerbaidzhan" [Title/abstract] OR "Jordan*" [Title/abstract] OR "Kazakh*" [Title/abstract] OR "Kazakstan*" [Title/abstract] OR "Syria*"[Title/abstract] OR "Yemen*" [Title/abstract] OR "Uzbek*" [Title/abstract] OR "Saudi Arabia*" [Title/abstract] OR "Afghanistan" [Title/abstract] OR "Iraq*" [Title/abstract] OR "Iran*" [Title/abstract] OR "Persia*" [Title/abstract] OR "Pakistan*" [Title/abstract] OR “Turkey” [Title/abstract] OR “Turk*”[Title/abstract] OR “Palestine” [Title/abstract] OR “Cyprus” [Title/abstract]	1,744,749
#4	#2 OR 3	2,029,122
#5	#1 AND #4 (filters from 2006-2023; English only)	1664

EMBASE, **6 Nov 2023**

No	Search term	No
#1	'implementation science'/exp/mj	1,905
#2	'implementation science*':ab,ti,kw OR 'implementation research':ab,ti,kw OR 'knowledge translation':ab,ti,kw OR 'knowledge transfer':ab,ti,kw OR ‘translational research’:ab,ti,kw OR ‘Dissemination and implementation research’:ab,ti,kw OR ‘D&I research’:ab,ti,kw	38,145
#3	‘Asia’/exp OR ‘China’/exp OR ‘Hong Kong’/exp OR ‘Japan’/exp OR ‘Korea’/exp OR ‘Taiwan’/exp OR ‘Mongolia’/exp OR ‘Bangladesh’/exp OR ‘Bhutan’/exp OR ‘India’/exp OR ‘Nepal’/exp OR ‘Sri Lanka’/exp OR ‘Brunei’/exp OR ‘Myanmar’/exp OR ‘Cambodia’/exp OR ‘Indonesia’/exp OR ‘Laos’/exp OR ‘Philippines’/exp OR ‘Malaysia’/exp OR ‘Singapore’/exp OR ‘Thailand’/exp OR ‘Timor-Leste’/exp OR ‘Vietnam’/exp OR ‘Macau’/exp OR ‘Bahrain’/exp OR ‘Armenia’/exp OR ‘Qatar’/exp OR ‘Georgia’/exp OR ‘Kuwait’/exp OR ‘Oman’/exp OR ‘Turkmenistan’/exp OR	1,413,536

	‘Lebanon’/exp OR ‘Kyrgyzstan’/exp OR ‘Israel’/exp OR ‘Tajikistan’/exp OR ‘United Arab Emirates’/exp OR ‘Azerbaijan’/exp OR ‘Jordan’/exp OR ‘Kazakhstan’/exp OR ‘Syria’/exp OR ‘Yemen’/exp OR ‘Uzbekistan’/exp OR ‘Saudi Arabia’/exp OR ‘Afghanistan’/exp OR ‘Iraq’/exp OR ‘Iran’/exp OR ‘Pakistan’/exp OR ‘Cyprus’/exp OR ‘Turkey’/exp OR ‘Maldives’/exp	
#4	**'asia*'**:ab,ti,kw OR **'china'**:ab,ti,kw OR **'chinese'**:ab,ti,kw OR **'hong kong'**:ab,ti,kw OR **'japan*'**:ab,ti,kw OR **'korea*'**:ab,ti,kw OR **'taiwan*'**:ab,ti,kw OR **'mongolia*'**:ab,ti,kw OR **'bangla*'**:ab,ti,kw OR **'bhutan*'**:ab,ti,kw OR **'india*'**:ab,ti,kw OR **'maldiv*'**:ab,ti,kw OR **'nepal*'**:ab,ti,kw OR **'sri lanka*'**:ab,ti,kw OR **'brunei*'**:ab,ti,kw OR **'myanmar*'**:ab,ti,kw OR **'burm*'**:ab,ti,kw OR **'cambodia*'**:ab,ti,kw OR **'indonesia*'**:ab,ti,kw OR **'laos'**:ab,ti,kw OR **'laotian*'**:ab,ti,kw OR **'lao pdr'**:ab,ti,kw OR **'philippin*'**:ab,ti,kw OR **'phillippin*'**:ab,ti,kw OR **'filipin*'**:ab,ti,kw OR **'malay*'**:ab,ti,kw OR **'singapore*'**:ab,ti,kw OR **'thai*'**:ab,ti,kw OR **'timor leste*'**:ab,ti,kw OR **'timor*'**:ab,ti,kw OR **'timor-leste*'**:ab,ti,kw OR **'east timor'**:ab,ti,kw OR **'vietnam*'**:ab,ti,kw OR **'viet nam'**:ab,ti,kw OR **'macau*'**:ab,ti,kw OR **'macao*'**:ab,ti,kw OR **'macanese*'**:ab,ti,kw OR **'bahrain*'**:ab,ti,kw OR **'armenia*'**:ab,ti,kw OR **'qatar*'**:ab,ti,kw OR **'georgia*'**:ab,ti,kw OR **'kuwait*'**:ab,ti,kw OR **'oman*'**:ab,ti,kw OR **'palestin*'**:ab,ti,kw OR **'turkmen*'**:ab,ti,kw OR **'lebanon'**:ab,ti,kw OR **'leban*'**:ab,ti,kw OR **'kyrgyz*'**:ab,ti,kw OR **'israel*'**:ab,ti,kw OR **'tajikistan'**:ab,ti,kw OR **'tadzhikistan'**:ab,ti,kw OR **'tajik*'**:ab,ti,kw OR **'united arab emirates'**:ab,ti,kw OR **'emirat*'**:ab,ti,kw OR **'uae'**:ab,ti,kw OR **'azerbaijan*'**:ab,ti,kw OR **'azerbaidzhan'**:ab,ti,kw OR **'jordan*'**:ab,ti,kw OR **'kazakh*'**:ab,ti,kw OR **'kazakstan*'**:ab,ti,kw OR **'syria*'**:ab,ti,kw OR **'yemen*'**:ab,ti,kw OR **'uzbek*'**:ab,ti,kw OR **'saudi arabia*'**:ab,ti,kw OR **'afghanistan'**:ab,ti,kw OR **'iraq*'**:ab,ti,kw OR **'iran*'**:ab,ti,kw OR **'persia*'**:ab,ti,kw OR **'pakistan*'**:ab,ti,kw OR **'turkey'**:ab,ti,kw OR **'turk*'**:ab,ti,kw OR **'palestine'**:ab,ti,kw OR **'cyprus'**:ab,ti,kw	2,360,230
#5	#1 OR #2	38,500
#6	#3 OR #4	2,667,102
#7	#5 AND #6	2,459
#8	#7 AND (**2006**:py OR **2007**:py OR **2008**:py OR **2009**:py OR **2010**:py OR **2011**:py OR **2012**:py OR **2013**:py OR **2014**:py OR **2015**:py OR **2016**:py OR **2017**:py OR **2018**:py OR **2019**:py OR **2020**:py OR **2021**:py OR **2022**:py OR **2023**:py) AND (**'article'**/it OR **'article in press'**/it)	1300

CINAHL, **6 Nov 2023**

No	Search term	No
S1	MM "Implementation Science"	625
S2	(TI “implementation science” OR AB “implementation science”) OR (TI “implementation research” OR AB “implementation research”) OR (TI “Knowledge transfer” OR AB “Knowledge transfer”) OR (TI “knowledge translation” OR AB “knowledge translation”) OR (TI “translational research” OR AB “translational research”) OR (TI “Dissemination and implementation research” OR AB “Dissemination and implementation research”) OR (TI “D&I research” OR AB “D&I research”)	9,753
S3	(MH "Asia") OR (MH "China") OR (MH "Hong Kong") OR (MH "Japan") OR (MH "Korea") OR (MH "Taiwan") OR (MH "Mongolia") OR (MH "Bangladesh") OR (MH "Bhutan") OR (MH "India") OR (MH "Nepal") OR (MH "Sri Lanka") OR (MH "Brunei") OR (MH "Myanmar") OR (MH "Cambodia") OR (MH "Indonesia") OR (MH "Laos") OR	349,740

	(MH "Philippines") OR (MH "Malaysia") OR (MH "Singapore") OR (MH "Thailand") OR (MH "Timor-Leste") OR (MH "Vietnam") OR (MH "Macau") OR (MH "Bahrain") OR (MH "Armenia") OR (MH "Qatar") OR (MH "Georgia") OR (MH "Kuwait") OR (MH "Oman") OR (MH "Turkmenistan" OR (MH "Lebanon") OR (MH "Kyrgyzstan") OR (MH "Israel") OR (MH "Tajikistan") OR (MH "United Arab Emirates") OR (MH "Azerbaijan") OR (MH "Jordan") OR (MH "Kazakhstan") OR (MH "Syria") OR (MH "Yemen") OR (MH "Uzbekistan") OR (MH "Saudi Arabia") OR (MH "Afghanistan") OR (MH "Iraq") OR (MH "Iran") OR (MH "Pakistan") OR (MH "Cyprus") OR (MH "Turkey") OR (MH "Maldives)	
S4	TI (Asia* OR China OR Chinese OR Hong Kong OR Japan* OR Korea* OR Taiwan* OR Mongolia* OR Bangla*OR Bhutan* OR India* OR Maldiv* OR Nepal* OR Sri Lanka* OR Brunei* OR Myanmar* OR Burm* OR Cambodia* OR Indonesia* OR Laos OR Lao People's Democratic Republic OR Laotian* OR Lao PDR OR Philippin*OR Phillippin* OR Filipin* OR Malay* OR Singapore* OR Thai* OR Timor Leste* OR Timor* OR Timor-Leste* OR East Timor OR Vietnam* OR Viet Nam OR Macau* OR Macao* OR Macanese* OR Bahrain* OR Armenia* OR Qatar* OR Georgia* OR Kuwait* OR Oman* OR Palestin* OR Turkmen* OR Lebanon OR Leban* OR Kyrgyz* OR Israel* OR Tajikistan OR Tadzhikistan OR Tajik* OR United Arab Emirates OR Emirat* OR UAE OR Azerbaijan* OR Azerbaidzhan OR Jordan* OR Kazakh* OR Kazakstan* OR Syria*OR Yemen* OR Uzbek* OR Saudi Arabia* OR Afghanistan OR Iraq* OR Iran* OR Persia* OR Pakistan* OR Turkey OR Turk*OR Palestine OR Cyprus)<br><br>OR AB (Asia* OR China OR Chinese OR Hong Kong OR Japan* OR Korea* OR Taiwan* OR Mongolia* OR Bangla*OR Bhutan* OR India* OR Maldiv* OR Nepal* OR Sri Lanka* OR Brunei* OR Myanmar* OR Burm* OR Cambodia* OR Indonesia* OR Laos OR Lao People's Democratic Republic OR Laotian* OR Lao PDR OR Philippin*OR Phillippin* OR Filipin* OR Malay* OR Singapore* OR Thai* OR Timor Leste* OR Timor* OR Timor-Leste* OR East Timor OR Vietnam* OR Viet Nam OR Macau* OR Macao* OR Macanese* OR Bahrain* OR Armenia* OR Qatar* OR Georgia* OR Kuwait* OR Oman* OR Palestin* OR Turkmen* OR Lebanon OR Leban* OR Kyrgyz* OR Israel* OR Tajikistan OR Tadzhikistan OR Tajik* OR United Arab Emirates OR Emirat* OR UAE OR Azerbaijan* OR Azerbaidzhan OR Jordan* OR Kazakh* OR Kazakstan* OR Syria*OR Yemen* OR Uzbek* OR Saudi Arabia* OR Afghanistan OR Iraq* OR Iran* OR Persia* OR Pakistan* OR Turkey OR Turk*OR Palestine OR Cyprus)	
S5	S1 AND S2	10,040
S6	S3 AND S4	542,732
S7	S5 AND S6	542
S8	**Limiters** - Published Date: 20060101-20231231<br>**Expanders** - Apply equivalent subjects<br>**Narrow by Language:** - english<br>**Search modes** - Find all my search terms	528

PsycINFO, 6 Nov 2023

No	Search term	No
#1	(implementation science OR implementation research OR knowledge translation OR knowledge transfer OR translational research OR dissemination and implementation research OR D&I research).ab,ti.	1021
#2	(Asia* OR China OR Chinese OR Hong Kong OR Japan* OR Korea* OR Taiwan* OR Mongolia* OR Bangla*OR Bhutan* OR India* OR Maldiv* OR Nepal* OR Sri Lanka* OR	313,619

	Brunei* OR Myanmar* OR Burm* OR Cambodia* OR Indonesia* OR Laos OR Lao People’s Democratic Republic OR Laotian* OR Lao PDR OR Philippin*OR Phillippin* OR Filipin* OR Malay* OR Singapore* OR Thai* OR Timor Leste* OR Timor* OR Timor-Leste* OR East Timor OR Vietnam* OR Viet Nam OR Macau* OR Macao* OR Macanese* OR Bahrain* OR Armenia* OR Qatar* OR Georgia* OR Kuwait* OR Oman* OR Palestin* OR Turkmen* OR Lebanon OR Leban* OR Kyrgyz* OR Israel* OR Tajikistan OR Tadzhikistan OR Tajik* OR United Arab Emirates OR Emirat* OR UAE OR Azerbaijan* OR Azerbaidzhan OR Jordan* OR Kazakh* OR Kazakstan* OR Syria*OR Yemen* OR Uzbek* OR Saudi Arabia* OR Afghanistan OR Iraq* OR Iran* OR Persia* OR Pakistan* OR Turkey OR Turk*OR Palestine OR Cyprus).ab,ti.	
#3	#1 AND #2	58
#4	#3 (Filter year: 2006-2023; peer reviewed journal)	52
