# Supplementary material for: Barriers to conducting implementation science research in Asia: An online survey
Source: PLoS One. 2025 Jun 26;20(6):e0325372. doi: 10.1371/journal.pone.0325372 (PMC12200828; doi:10.1371/journal.pone.0325372)
Supplement: Attachment — Submitted filename: Response to reviewer_20.4.2025.docx [file pone.0325372.s004.docx]

**Reviewer 1**

| **No** | **Comment** | **Response to reviewer** |
| --- | --- | --- |
| 1. | Although the intro section discusses barriers to IS research in Asia, explicit justification for why this particular study is necessary is visibly missing. The author should justify clearly why this study. | We have now provided a justification on why this study should be conducted.  Page 2  *It has been 17 years since IS emerged, with the launch of the first dedicated specialist scientific journal, Implementation Science in 2006 [6]. However, although IS research in Asia is still limited, it is gradually gaining traction [17]. Based on narrative reviews and commentaries, the slow adoption of IS research in Asia is said to be attributed to the lack of emphasis by policymakers in setting research agendas or fund implementation research, the lack of applicability of IS theory/model/framework in low and middle resource settings, the monopoly of knowledge sharing by high-income countries, the lack of emphasis of equity and community participation in the implementation research agenda, and the lack of IS expertise and basic understanding of IS [18, 19].* ***While there has been a call for increasing IS research in the Asia [18], there is a lack of initiatives in advancing the field in the region. Hence, understanding barriers to conducting IS research can help to raise awareness on the barriers, and to identify potential strategies to promote the conduct of IS research further. Currently, understanding of barriers to IS research in the Asian settings has not been empirically studied. Therefore, this study aims to assess barriers to conducting IS research in Asia.*** |
| 2. | The authors try to share literature on IS research in high-income countries but do not compare same in other LMICs beyond Asia. At least, a broader contextualization by briefly mentioning IS research in another continent say in Africa or Latin America, would strengthen the argument. | We have now provided information on the status of IS research in the Africa region.  Page 2  *However, most of the IS literature are from Western high-income countries such as the USA, UK, Australia, and Canada [8-10].* ***Nevertheless, some IS work has been observed in low- and middle-income countries particularly from the Africa region including adaption of IS frameworks, and selection and implementation of strategies [11, 12]. The advancement of IS research in the African region can be attributed to the support by Western-country partners or international organisation, which provided funding, and cross-country learning opportunities for IS training and capacity building in the region [13, 14], although the lack of funding, collaborators and mentorship prevailed as main barriers to engage in IS research [14].*** |
| 3. | The authors clearly state that a cross-sectional online survey was used but do not justify why this design was chosen over the others. Also, the authors should briefly explain why an online survey is the most appropriate method for this study and a continent in the global south where online-related issues can sometimes be a challenge. | The reason why we chose a cross-sectional online survey was because of the wide study scope that involves the Asian region. Online survey is also cost effective and provide convenience for participants who may be from different time zones to complete the survey anytime, and anywhere.  While internet access can be an issue for some participants in the region to participate in this study, we believe that this will not be a major barrier as the target participants of this study are scientific researchers where the nature of their work requires them to have Internet access. Furthermore, as this study focusses on the Asian region, we assumed that most of the target participants would be residing in the Asian region where the internet penetration in this region is more than 60% in 2024. Nevertheless, we have now included this point in the Limitation section.  We have now included the information above in the manuscript at:  Page 3  **Methodology**  *Study design*  *This study adopted a cross-sectional online survey design.* ***This study design was chosen to provide current insights on the various barriers to conducting IS research in the Asian region. The wide study scope that involves the Asian region necessitates the use of online survey as it is cost effective and provide convenience for participants who may be from different time zones to complete the survey anytime, and anywhere.***  Page 15  **Strengths and limitations**  *One notable strength of this study is that it is the first to examine the challenges associated with conducting implementation science research in Asian contexts. Secondly, this study assessed barriers to conducting IS research in Asia among participants who have conducted IS research in the Asian settings. The findings presented reflect real-life and not perceived barriers. The limitations of this study include: the sampling frame is limited to researchers who has published their IS research in journals, hence, the findings presented may reflect those who primarily worked in the academic settings and may not be generalisable to IS researchers from non-academic or clinical-settings; whose work are usually not visible to scientific community.* ***As with any online survey, the response rate for this study is low. Although Internet access could pose a barrier for some participants, it is unlikely to be an issue for the target participants of this study, as the nature of their work typically requires them to have Internet access. Furthermore, since this study concentrates on the Asian region, it is presumed that the majority of the target participants will be from this area, where internet penetration exceeded 60% in 2024 [45]. The small sample size in this study rendered insufficient power to draw significant conclusions from the findings. Nevertheless, the descriptive findings provide valuable insights into barriers to conducting IS in the Asian settings.*** |
| 4. | Participant Response Rate and Bias Consideration  The authors should briefly include a statement about the response rate and whether any follow-up measures were taken, like whether reminders were sent to encourage participation. This will help improve the bias considerations in the paper.  The authors should clearly indicate how the questionnaires or surveys were administered. Was it through emails, a web-based platform as well as a narration on how security protection was ensured for electronic data? For example, if emails were used, did authors use their institution email addresses or personal email addresses etc? | We have provided information in the participant response rate at the Results section, and included information on the follow-up measures in the Data Collection Process. The response rate at each email reminder can be found in Figure 1. Information on how the questionnaire was administered has also been provided at the Methodology section.  We have now made it clearer in the manuscript on how the questionnaire was administered, how security protection was ensured for electronic data, and how the participants’ email addresses were obtained in the Data Collection Process section.  Page 6  *Instrument*  *The questionnaire in this study was adapted from a study conducted in the US on barriers towards engagement in IS research [22, 23], literature review on barriers to conducting implementation science research [18, 24-27], and expert input from four healthcare researchers who have conducted implementation science research in Asia. The questionnaire was sent to them with a rating scale on the relevance for each of the questionnaire item. They were also asked to provide comments in terms of clarity, and the significance and completeness of response options. Minor changes were made to the questionnaire such as revision of sentences that were unclear, and language and grammatical errors.* ***The questionnaire was in English language and designed to be completed online using the RedCap electronic data capture tools hosted at the Universiti Malaya [28, 29].***  *Data collection process*  *The data collection for this study was conducted from April to June 2024.* ***Participants were invited to participate in this study through email, where the RedCap survey link was included.*** ***Given that the participants were researchers who had conducted IS research in Asian settings as evidenced in the papers retrieved, their email address as a corresponding author was used. For participants who were not corresponding author, their email address was sought through Google search based on their affiliations given in the papers.*** *The participant information sheet and the informed consent was embedded as part of the electronic questionnaire.* ***Data confidentiality was ensured by informing participants, through the participant information sheet, that their data would be stored on the researcher’s institution’s RedCap server, which provides a secure data storage environment.*** *The participant ticked and dated the latest approved version of the informed consent form to proceed with answering the questionnaire.*  ***Following the initial invitation email, two reminders were sent out: the first one a week later and the second one two weeks after the original invitation. If there were no responses after the second reminder, invitation was sent to the first or last authors, who were not corresponding authors of the study. Two reminders were also given. The online data collection process is illustrated in Figure 1.*** |
| 5. | The paper indicates that a 40.1% response rate was recorded. With this % it is easy for readers to perceive that the % of the non-respondents could affect the findings. So, the authors should briefly indicate where the non-respondents could have affected the findings. | We have highlighted the low response rate as a study limitation.  Page 15  **Strengths and limitations**  *One notable strength of this study is that it is the first to examine the challenges associated with conducting implementation science research in Asian contexts. Secondly, this study assessed barriers to conducting IS research in Asia among participants who have conducted IS research in the Asian settings. The findings presented reflect real-life and not perceived barriers. The limitations of this study include: the sampling frame is limited to researchers who has published their IS research in journals, hence, the findings presented may reflect those who primarily worked in the academic settings and may not be generalisable to IS researchers from non-academic or clinical-settings; whose work are usually not visible to scientific community.* ***As with any online survey, the response rate for this study is low.*** *While Internet access may be a barrier for some participants to participate in this study, this may not be the case given the target participants of this study where the nature of their work would require them to have Internet access. Furthermore, as this study focusses on the Asian region, it is assumed most of the target participants would be from the Asian region where the internet penetration in this region is more than 60% in 2024 [45].* ***The small sample size in this study rendered insufficient power to draw significant conclusions from the findings. Nevertheless, the descriptive findings provide valuable insights into barriers to conducting IS in the Asian settings.*** |
| 6. | Results and Discussion  I think the authors should advance the Statistical Analysis: The bivariate analysis is relatively basic. More advanced statistical techniques could have been used to explore relationships between key variables.  **Also, there is the absence of the use of numbers in the discussion. Authors should use a few numbers, or percentages to enhance the comparison hence strengthening the low patronage of IS in Asia.** The numbers must speak to the barriers and the context.  Though the quotes are good as stated in Table 2, the strength of the paper will improve if the authors **could add the % of participants who mentioned each barrier.** | Unfortunately, given the small sample size of this study and the small number across sub-groups, we were unable to draw significant conclusions from the findings. We have taken the advice by the second reviewer to report the findings descriptively as non-significant findings can also provide important insights into the barriers to conduct IS research. We have included this point as a study limitation. Page 15 Strengths and limitations … As with any online survey, the response rate for this study is low. While Internet access may be a barrier for some participants to participate in this study, this may not be the case given the target participants of this study where the nature of their work would require them to have Internet access. Furthermore, as this study focusses on the Asian region, it is assumed that most of the target participants would be from the Asian region where the internet penetration in this region is more than 60% in 2024 [44]. **The small sample size in this study rendered insufficient power to draw significant conclusions from the findings. Nevertheless, the descriptive findings provide valuable insights into barriers to conducting IS in the Asian settings.** The use of percentages are now included in the Discussions. Page 14 ***Discussions*** *To our knowledge, this is the first study that assessed the barriers to conducting IS research in Asia. The study findings indicate that IS research is gaining momentum in the Asian region as many of the participants reported to have perform or collaborated on a study examining the translation of an intervention into routine settings in the past five years. Nevertheless, there was a high proportion who reported barriers to performing IS research.* ***The prevalence of barriers reported in this study are higher compared to other studies conducted in the Western countries where the most prevalent barrier reported was the lack of funding (Asia: 86.7%; USA: 82.7%; multicountry study (Australia, Netherlands, Canada, USA, and the UK): 17.3%) followed by the lack of awareness (Asia: 59.5%; USA: 50.9%; multicountry study (Australia, Netherlands, Canada, USA, and the UK): 0.8%), and the lack of organisational support (Asia: 48.7%; USA: 28.9%; multicountry study (Australia, Netherlands, Canada, USA, and the UK): 9%) [22, 24]. These barriers were also echoed in another study conducted in Western Europe countries namely Germany, Austria and Switzerland [25].*** *This study also found that many of the IS research was conducted among countries in South Asia specifically India, Bangladesh, and Nepal, while many reported to received funding from non-Asian countries such as from the UK and US.* We have now provided the % of participants who mentioned each of the barrier in Table 2. Page 13 **Table 2: Additional barriers (n=52)** **Category** \| **Selected quotes**; Lack of capacity or training support **(17.3%, n=9)** \| - Weak capacities in the developing country governments to actively participate in the design, implementation, and dissemination of implementation science research, and to apply the learning for policy and programme improvements. - Lack of a critical mass and not having IS topic as core public health education.; Lack of commitment and involvement from stakeholders other than researchers such as senior leaders, clinicians, or the government **(15.4,%, n=8)** \| - Resistance and poor understanding from stakeholders other than researchers regarding the multi-faceted efforts required for successful implementation, e.g. policy makers, management within health institutions. - Difficulty in getting access to healthcare organisations to do the research. - Lack of collaboration and support from the government. The slow and difficult process to work with government stakeholders.; Perceived low value of IS research (**11.5%, n=6)** \| - IS research is often not viewed as 'cutting edge' or transformational research among colleagues, academic institutions, and funding organisations. - Studies focused on neurosciences, genetics, or artificial intelligence sound fancy while implementation science is not.; Existing knowledge, system and practices challenge **(5.8%, n=3)** \| - Getting support in real setting health care system is very difficult because it already has system and interventions - As an applied field that draws on multiple disciplines and methodologies, implementation research grant applications often get stuck with purist and pedantic discipline-oriented peer reviewers.; IS lack of scientific rigour **(3.9%, n=2)** \| - IS often heavily relies on a qualitative approach, which many consider fluffy science. It lacks quantitative methods.; Lack of academic progression indicators **(1.9%, n=1)** \| - Translating research into practice often results in outputs that do not add to academic's career progression like a publication in a peer-reviewed journal would. It takes a lot more effort and time to do an implementation science activity versus doing a secondary data analysis that would lead to a peer-reviewed publication. |
| 7. | The Conclusion is very short and lacks a statement on IS and its importance in research or practice | We have now expanded the Conclusion and provided more descriptions about IS and its importance in research and practice in the Asian region.  Page 16  **Conclusion**  *This study sheds light on the multifaceted barriers hindering the conduct of IS research in Asia, revealing a pressing need for systemic change to enable its advancement. The findings highlight that while IS research is gradually gaining traction in the region, it remains significantly constrained by limited funding, low awareness, insufficient training opportunities, and inadequate stakeholder engagement. Additionally, challenges such as the perceived low value of IS, lack of academic incentives, and systemic rigidity further impede progress. These barriers suggest that to advance IS in Asia, concerted efforts are required to establish dedicated funding streams, embed IS education within academic curricula, develop regional training programmes, and foster strategic, long-term collaborations with key stakeholders. Addressing these issues will not only build research capacity but also enable the generation of contextually relevant evidence to improve health outcomes and bridge the research-to-practice gap in diverse Asian healthcare settings.* |

**Reviewer 2**

| **No** | **Comment** | **Response to reviewer** |
| --- | --- | --- |
| 1. | Please include a more substantial background stating that some work has been done on adapting the frameworks and ERIC strategies to the low-middle income context. For example:  • Means, A.R., Kemp, C.G., Gwayi-Chore, MC. et al. Evaluating and optimizing the consolidated framework for implementation research (CFIR) for use in low- and middle-income countries: a systematic review. Implementation Sci 15, 17 (2020). <https://doi.org/10.1186/s13012-020-0977-0>  • Lovero, K.L., Kemp, C.G., Wagenaar, B.H. et al. Application of the Expert Recommendations for Implementing Change (ERIC) compilation of strategies to health intervention implementation in low- and middle-income countries: a systematic review.Implementation Sci 18, 56 (2023). <https://doi.org/10.1186/s13012-023-01310-2>  • Kabir Sheikh, James Hargreaves, Mishal Khan, Sandra Mounier-Jack, Implementation research in LMICs—evolution through innovation, Health Policy and Planning, Volume 35, Issue Supplement_2, November 2020, Pages ii1–ii3, <https://doi.org/10.1093/heapol/czaa118> | We have now included information on the work done in the LMIC region, and cited two references given.  Page 2  *Implementation science (IS) is a field of study that can help to bridge the knowledge-practice gap. It is defined as “the scientific study of methods to promote the systematic uptake of research findings and other evidence-based practices into routine practice, and hence, to improve the quality and effectiveness of health services” [6]. In the last decade, the field has advanced rapidly and a wide range of research studies have been conducted including exploring barriers and facilitators to implementation, exploring influences on the provider, patient,* *and organizational behaviours, testing implementation strategies’ effectiveness, developing theories and frameworks to support implementation, and developing and validating measures to assess implementation outcomes [7]. However, most of the IS literature are from Western high-income countries such as the USA, UK, Australia, and Canada [8-10].* ***Nevertheless, some IS work has been observed in low- and middle-income countries particularly from the Africa region including the adaptation of IS frameworks and the selection and application of implementation strategies [11, 12]. The advancement of IS research in the African region can be attributed to the support by Western-country partners or international organisation, which provided funding, and cross-country learning opportunities for IS training and capacity building in the region [13, 14], although the lack of funding, collaborators and mentorship prevailed as main barriers to engage in IS research [14].*** |
| 2. | On page 2, at the end of the introduction, the authors say, “The findings of this study will inform the development of strategies to promote IS research and ultimately increase the use of evidence-based interventions in the region.”  The second part of this sentence is not a direct outcome of this study, so it sounds far-fetched. I recommend stating the direct implications of the findings, for example, demonstrating the challenges, raising awareness of the barriers, and ultimately identifying potential strategies to promote IS research further. | Thank you for the suggestion. We have now revised the sentence based on the reviewer’s suggestion as follows:  Page 2-3  *It has been 17 years since IS emerged, with the launch of the first dedicated specialist scientific journal, Implementation Science in 2006 [6]. However, although IS research in Asia is still limited, it is gradually gaining traction [17]. Based on narrative reviews and commentaries, the slow adoption of IS research in Asia is said to be attributed to the lack of emphasis by policymakers in setting research agendas or fund implementation research, the lack of applicability of IS theory/model/framework in low and middle resource settings, the monopoly of knowledge sharing by high-income countries, the lack of emphasis of equity and community participation in the implementation research agenda, and the lack of IS expertise and basic understanding of IS [18, 19]. While there has been a call for increasing IS research in the Asia [18], there is a lack of initiatives in advancing the field in the region.* ***Hence, understanding barriers to conducting IS research can increase awareness and help to identify strategies to enhance its development. Currently, understanding of barriers to IS research in the Asian settings has not been empirically studied. Therefore, this study aims to assess barriers to conducting IS research in Asia****.* |
| 3. | On page 3, the identification of the sample is not very clear. | Thank you for pointing this out. We have now made the identification of the sample clearer in the manuscript.  Page 3  *Participants*  *Researchers who had conducted IS research in Asian settings were invited to participate in this study.* ***These participants were identified from implementation science studies that were published, whereby the corresponding authors of the studies were invited to participate.*** *The study sampling frame was obtained via an online literature search on IS research conducted in Asian settings using the PubMed, EMBASE, CINAHL, and PsycINFO databases. More information on the search process and strategy can be found in Appendix 1.*  *The inclusion criteria for the articles were:*   - *study that use an implementation theory, model, framework in guiding the implementation effort [1], perform a process or implementation evaluation, or described use of any of the 73 implementation strategies (ERIC), or presented findings on implementation outcomes (i.e., acceptability, adoption, appropriateness, feasibility, fidelity, implementation cost, penetration and sustainability) [2]* - *conducted in Asian settings* - *studies conducted by non-Asian researchers, but the study was conducted in an Asian setting* - *original research (rather than a letter, commentary, protocol, editorial, review)* - *was authored by a named (rather than an anonymous) author* - *published from 2006 onwards* - *published in English*   *The exclusion criteria were:*   - *studies conducted in multi-countries (including both Asian and non-Asian countries).* - *studies conducted by Asian researchers but in non-Asian settings.*   *From the literature search, a total of 2295 articles were retrieved. These articles were screened by title and abstract by the two researchers of this study (WTT, CYO). Articles assessed were included or excluded according to the inclusion and exclusion criteria. Once the researchers finished their screening, they cross-checked each other’s assessment for correctness. Discrepancies were resolved through discussions. From the 2295 articles, 2013 articles were removed as they did not fit the study inclusion criteria.* ***The final number of articles included in this study were 282, and the corresponding authors’ email addresses were extracted.*** *Figure 1 illustrates the process of participant identification and the data collection process of this study.* |
| 4. | On page 4, the authors state, “The questionnaire in this study was adapted from a study conducted in the US on barriers towards engagement in IS research [18, 19], literature review, and input from healthcare researchers who have conducted implementation science research in Asia.”  It would be helpful to describe what kind of literature review was conducted or to cite the manuscripts included in the review, how many researchers provided input, how that input was gathered, and what changes were made to the instrument based on the feedback. It is unclear if the input is the same as that from the pilot test involving 19 researchers. | We have now revised the text to make it clearer on how the questionnaire for this study was developed. We have now provided information on the literature review that was conducted and cited the manuscripts included in the review. The expert input provided by healthcare researchers is different from the pilot testing of the questionnaire, which involved 19 researchers. We have now made this point clearer in the manuscript. Information on how many researchers provided input, how that input was gathered, and what changes were made to the instrument based on the feedback are now included.  Page 6  *Instrument*  ***The questionnaire in this study was adapted from a study conducted in the US on barriers towards engagement in IS research [22, 23], literature review on barriers to conducting implementation science research [18, 24-27], and expert input from four healthcare researchers who have conducted implementation science research in Asia. The questionnaire was sent to them with a rating scale on the relevance for each of the questionnaire item. They were also asked to provide comments in terms of clarity, and the significance and completeness of response options. Minor changes were made to the questionnaire such as revision of sentences that were unclear, and language and grammatical errors. The questionnaire was in English language and designed to be completed online using the RedCap electronic data capture tools hosted at the Universiti Malaya [28, 29].***    ***Subsequently, the questionnaire was pilot tested with 19 healthcare researchers who have experience with implementation research before finalising for actual study. No changes were made to the questionnaire.*** *The finalised version of the questionnaire comprised of items that examine socio-demographic characteristics, implementation science research practices, and yes or no items which examines four barriers to conducting implementation science research that is, the lack of awareness, funding, time, and organisational support. An open-ended question was provided at the end of the questionnaire to gather additional information on barriers to IS research engagement that was not covered in the quantitative items.* |
| 5. | On page 4, in the data analysis subsection, please provide details on how the qualitative analysis was conducted. For instance, how many comments were there? Was coding performed to identify categories? How many researchers analyzed the qualitative data? How were any discrepancies resolved? How many new types of barriers were identified through qualitative analysis? | We have now provided more information on how the qualitative data analysis was conducted. Information on the number of comments, and researchers involved in the qualitative data analysis, the number of comments, how discrepancies were resolved, and how many new types of barriers were identified from qualitative data analysis are now provided.  Page 6-7  *Data analysis*  *A descriptive analysis was conducted to summarize the results, utilizing frequencies and percentages with SPSS 28.0 (SPSS Inc, Chicago, IL) software.* ***Responses to open-ended questions were analysed through qualitative content analysis, where data was condensed into categories based on the emerging findings. Initially, codes were developed to specific data sections that represented their meaning. Then, similar codes were grouped together to form categories. The broader categories represent the additional barriers to conducting IS research. The qualitative analysis was conducted by the researcher (WTT), and the findings were cross-checked with the other two researchers (CYO; YEL) to ensure accuracy. Any discrepancies were resolved through discussions.***  Page 7  **Results**  *Analysis of the open-ended responses*  *In addition to the commonly recognized barriers to conducting IS research - such as limited funding, awareness, time, and organizational support -* ***open-ended responses revealed six additional challenges. These include insufficient commitment and engagement from stakeholders beyond researchers, a perceived lack of value attributed to IS research, concerns about the field’s scientific rigour, challenges arising from existing knowledge, systems, and practices, inadequate capacity or training opportunities, and the absence of clear indicators for academic progression within the field*** *(Table 2).* |
| 6. | Table 1: Given the small sample size across subgroups, I recommend presenting the results at a descriptive level and refraining from reporting associations, as these can lead to incorrect conclusions. For instance, a statistically significant association between the number of years of conducting health services research and barriers is notable, but it does not imply that other statistically non-significant associations are irrelevant. The study lacks sufficient power to draw such conclusions. | Thank you for the suggestion. We have now remove the reporting of associations and present the results at a more descriptive level. We have also now highlighted that the small sample rendered insufficient power to draw significant conclusions from the findings as a study limitation.  Page 11 - Refer to Table 1  **Table 1: Barriers to conducting IS research and socio-demographic characteristics and IS practice**  *Page 15*  ***Strengths and limitations***  *One notable strength of this study is that it is the first to examine the challenges associated with conducting implementation science research in Asian contexts. Secondly, this study assessed barriers to conducting IS research in Asia among participants who have conducted IS research in the Asian settings. The findings presented reflect real-life and not perceived barriers. The limitations of this study include: the sampling frame is limited to researchers who has published their IS research in journals, hence, the findings presented may reflect those who primarily worked in the academic settings and may not be generalisable to IS researchers from non-academic or clinical-settings; whose work are usually not visible to scientific community. As with any online survey, the response rate for this study is low. While Internet access may be a barrier for some participants to participate in this study, this may not be the case given the target participants of this study where the nature of their work would require them to have Internet access. Furthermore, as this study focusses on the Asian region, it is assumed that most of the target participants would be from the Asian region where the internet penetration in this region is more than 60% in 2024 [45].* ***The small sample size in this study rendered insufficient power to draw significant conclusions from the findings. Nevertheless, the descriptive findings provide valuable insights into barriers to conducting IS in the Asian settings.*** |
| 7. | For transparency and replicability of the study, please include the instrument to the supplemental file. | We have now included the questionnaire as a supplementary file. |
