# Supplementary material for: Barriers to conducting implementation science research in Asia: An online survey
Source: PLoS One. 2025 Jun 26;20(6):e0325372. doi: 10.1371/journal.pone.0325372 (PMC12200828; doi:10.1371/journal.pone.0325372)
Supplement: Attachment — Submitted filename: Reviewer Comments - IS.docx [file pone.0325372.s002.docx]

***Reviewer Comments***

Introduction

Gaps in Literature and Justification

Although the intro section discusses barriers to IS research in Asia, explicit justification for why this particular study is necessary is visibly missing. The author should justify clearly why this study.

Scope and Specificity

Th authors try to share literature on IS research in high-income countries but do not compare same in other LMICs beyond Asia. At least, a broader contextualization by briefly mentioning IS research in another continent say in Africa or Latin America, would strengthen the argument.

Methods

Lack of Justification for Study Design

The authors clearly state that a *cross-sectional online survey* was used but do not justify why this design was chosen over the others. Also, the authors should briefly explain why an online survey is the most appropriate method for this study and a continent in the global south where online-related issues can sometimes be a challenge.

Participant Response Rate and Bias Consideration

The authors should briefly include a statement about the response rate and whether any follow-up measures were taken, like whether reminders were sent to encourage participation. This will help improve the bias considerations in the paper. The authors should clearly indicate how the questionnaires or surveys were administered. Was it through emails, a web-based platform as well as a narration on how security protection was ensured for electronic data? For example, if emails were used, did authors use their institution email addresses or personal email addresses etc?

The paper indicates that a 40.1% response rate was recorded. With this % it is easy for readers to perceive that the % of the non-respondents could affect the findings. So, the authors should briefly indicate where the non-respondents could have affected the findings. Results and

Discussion

I think the authors should advance the Statistical Analysis: The bivariate analysis is relatively basic. More advanced statistical techniques could have been used to explore relationships between key variables. Also, there is the absence of the use of numbers in the discussion. Authors should use a few numbers, or percentages to enhance the comparison hence strengthening the low patronage of IS in Asia. The numbers must speak to the barriers and the context. Though the quotes are good as stated in Table 2, the strength of the paper will improve if the authors could add the % of participants who mentioned each barrier.

The Conclusion is very short and lacks a statement on IS and its importance in research or practice
